# Supplementary material for: Genome-Wide Transcriptomic Identification and Functional Insight of Lily WRKY Genes Responding to Botrytis Fungal Disease
Source: Plants (Basel). 2021 Apr 15;10(4):776. doi: 10.3390/plants10040776 (PMC8071302; doi:10.3390/plants10040776)
Supplement: Supplementary file 1 [file plants-10-00776-s001.zip › plants-1104701-supplementary/Table S1-S6 (Revised)-Apr 14.pdf]

Supplementary Table

Table S1: Molecular sequence characteristics of *LlWRKY* (Full CDS) gene family in *Lilium longiflorum*

Gene Name	Gene ID	CDS length (bp)	Amino acid length (aa)	Theoretical pI/Mw	Predicted TargetP location	Type of WRKY	PDB ID	Identity (%)	Score (E-value)	Species
LlWRKY1	MH614337	885	294	9.53/31811.21	Any other	IId	2AYD_A	55	4.00E-21	*A.thaliana*
LlWRKY2	MH614338	657	218	5.98/24236.28	Any other	IIe	5W3X_B	58	3.00E-20	*A.thaliana*
LlWRKY3	MH614339	1137	390	6.56/43520.00	Chloroplast	IC	1WJ2_A	77	8.00E-37	*A.thaliana*
LlWRKY4	MH614340	792	263	5.91/29225.39	Chloroplast	IN	1WJ2_A	54	1.00E-12	*A.thaliana*
LlWRKY5	MH614341	2157	718	6.26/78425.80	Any other	IC	1WJ2_A	86	2.00E-38	*A.thaliana*
LlWRKY6	MH614342	1896	631	5.54/68315.29	Any other	IC	1WJ2_A	86	7.00E-38	*A.thaliana*
LlWRKY7	MH614343	996	331	6.46/37108.24	Any other	IIc	1WJ2_A	67	3.00E-27	*A.thaliana*
LlWRKY8	MH614344	318	105	8.47/37108.24	Any other	IIa	1WJ2_A	43	1.00E-13	*A.thaliana*
LlWRKY9	MH614345	1596	531	7.74/57151.11	Chloroplast	IC	1WJ2_A	89	3.00E-40	*A.thaliana*
LlWRKY10	MH614346	1512	503	5.44/54026.08	Any other	IC	1WJ2_A	69	7.00E-30	*A.thaliana*
LlWRKY11	MH614347	462	153	8.93/17332.73	Any other	IIa	2AYD_A	53	6.00E-23	*A.thaliana*
LlWRKY12	MH614348	762	253	8.92/28701.72	Any other	IIa	2AYD_A	53	6.00E-23	*A.thaliana*
LlWRKY13	MH614349	963	320	6.01/36034.45	Any other	IIa	2AYD_A	62	1.00E-20	*A.thaliana*
LlWRKY14	MH614350	975	324	8.79/13446.32	Any other	III	5W3X_B	57	2.00E-19	*A.thaliana*
LlWRKY15	MH614351	348	115	7.76/35238.68	Any other	IIc	1WJ2_A	58	3.00E-27	*A.thaliana*
LlWRKY16	MH614352	558	184	5.74/20599.43	Any other	IIc	1WJ2_A	58	2.00E-26	*A.thaliana*
LlWRKY17	MH614353	927	308	4.75/34641.69	Any other	III	5W3X_B	50	6.00E-14	*A.thaliana*
LlWRKY18	MH614354	801	266	5.49/29109.49	Chloroplast	IIe	2AYD_A	57	2.00E-19	*A.thaliana*
LlWRKY19	MH614355	588	195	9.45/21874.96	Any other	III	5W3X_B	51	7.00E-15	*A.thaliana*
LlWRKY20	MH614356	579	192	9.91/21684.86	Any other	III	5W3X_B	55	7.00E-15	*A.thaliana*
LlWRKY21	MH614357	897	298	5.13/33608.85	Any other	III	5W3X_B	50	7.00E-18	*A.thaliana*
LlWRKY22	MH614358	1602	533	6.58/58591.83	Any other	IIb	2AYD_A	51	2.00E-20	*A.thaliana*
LlWRKY23	MH614359	840	279	5.69/30451.39	Any other	IIe	5W3X_B	53	7.00E-21	*A.thaliana*
LlWRKY24	MH614360	831	276	5.89/30107.93	Any other	IIe	5W3X_B	53	8.00E-21	*A.thaliana*

Table S2: Molecular sequence characteristics of *LlWRKY* (Partial CDS) gene family *in Lilium longiflorum*

Gene Name	Gene ID	CDS length (bp)	Amino acid length (aa)	Theoretical pI/Mw	Predicted TargetP location	Type of WRKY	PDB ID	Identity (%)	Score (E-value)	Species
LlWRKY25	MK452769	529	169	10.34/18981.15	Any other	IIc	1WJ2_A	58	2.00E-22	*A.thaliana*
LlWRKY26	MK452770	271	81	10.89/9401.07	Mitochondrion	IId	2AYD_A	46	2.00E-09	*A.thaliana*
LlWRKY27	MK452771	440	137	8.47/12322.88	Chloroplast	IIa	1WJ2_A	49	7.00E-07	*A.thaliana*
LlWRKY28	MK452772	920	285	6.42/32861.80	Any other	IN	1WJ2_A	81	2.00E-38	*A.thaliana*
LlWRKY29	MK452773	384	124	6.78/14569.50	Any other	IIc	1WJ2_A	57	3.00E-13	*A.thaliana*
LlWRKY30	MK452774	386	119	9.76/14048.66	Secretory pathway	IIc	2AYD_A	58	1.00E-11	*A.thaliana*
LlWRKY31	MK452775	441	142	10.46/16449.99	Any other	III	5W3X_B	50	1.00E-15	*A.thaliana*
LlWRKY32	MK452776	1096	339	8.92/39069.58	Any other	III	5W3X_B	50	3.00E-15	*A.thaliana*
LlWRKY33	MK452777	1055	327	9.03/37548.58	Any other	III	5W3X_B	53	8.00E-19	*A.thaliana*
LlWRKY34	MK452778	568	181	8.91/20929.72	Any other	IId	2AYD_A	46	2.00E-07	*A.thaliana*
LlWRKY35	MK452779	729	230	9.25/26367.66	Chloroplast	IIe	5W3X_B	61	3.00E-18	*A.thaliana*
LlWRKY36	MK452780	976	314	11.26/35702.80	Secretory pathway	IIe	5W3X_B	56	9.00E-22	*A.thaliana*
LlWRKY37	MK452781	269	86	9.33/9888.85	Secretory pathway	IIb	2AYD_A	61	5.00E-21	*A.thaliana*
LlWRKY38	MK452782	646	215	8.61/24625.81	Any other	III	5W3X_B	55	4.00E-13	*A.thaliana*

Table S3: The identified LlWRKY proteins in *Lilium longiflorum* and their putative orthologous gene in rice.

Genes	Orthologous genes	Genes ID
LlWRKY1	OsWRKY68, 13, 39, 14, 17	DAA05133.1, ABO34049.1, DAA05104.1, DAA05079.1
LlWRKY2	OsWRKY68, 13, 39, 14, 17	DAA05133.1, ABO34049.1, DAA05104.1, DAA05079.1, ABR25636.1, DAA05087.1 DAA05084.1, AAT84161.1
LlWRKY3	OsWRKY55, 22, 19, 74, 71, 28, 32, 53	ABR25636.1, DAA05087.1, DAA05084.1, AAT84161.1, Q6QHD1.1,Q0DAJ3.2, DAA05097.1, BAF75367.1
LlWRKY4	OsWRKY55, 22, 19, 74, 71, 28, 32, 53, 10	ABR25636.1, DAA05087.1, DAA05084.1, AAT84161.1, Q6QHD1.1, Q0DAJ3.2, DAA05097.1, BAF75367.1
LlWRKY5	OsWRKY55, 22, 19, 74, 71, 28, 32, 53	ABR25636.1, DAA05087.1, DAA05084.1, AAT84161.1, Q6QHD1.1, Q0DAJ3.2, DAA05097.1, BAF75367.1
LlWRKY6	OsWRKY55, 22, 19, 74, 71, 28, 32, 53	ABR25636.1, DAA05087.1, DAA05084.1, AAT84161.1, Q6QHD1.1, Q0DAJ3.2, DAA05097.1, BAF75367.1
LlWRKY7	OsWRKY36	DAA05101.1
LlWRKY8	OsWRKY68, 13, 39, 14, 17	DAA05133.1, ABO34049.1, DAA05104.1, DAA05079.1
LlWRKY9	OsWRKY55, 22, 19, 74, 71, 28, 32, 53	ABR25636.1, DAA05087.1, DAA05084.1, AAT84161.1, Q6QHD1.1, Q0DAJ3.2, DAA05097.1, BAF75367.1
LlWRKY10	OsWRKY55, 22, 19, 74, 71, 28, 32, 53	ABR25636.1, DAA05087.1, DAA05084.1, AAT84161.1, Q6QHD1.1, Q0DAJ3.2, DAA05097.1, BAF75367.1
LlWRKY11	OsWRKY55, 22, 19, 74, 71, 28, 32, 53	ABR25636.1, DAA05087.1, DAA05084.1, AAT84161.1, Q6QHD1.1, Q0DAJ3.2, DAA05097.1, BAF75367.1
LlWRKY12	OsWRKY55, 22, 19, 74, 71, 28, 32, 53	ABR25636.1, DAA05087.1, DAA05084.1, AAT84161.1, Q6QHD1.1, Q0DAJ3.2, DAA05097.1, BAF75367.1
LlWRKY13	OsWRKY55, 22, 19, 74, 71, 28, 32, 53	ABR25636.1, DAA05087.1, DAA05084.1, AAT84161.1, Q6QHD1.1, Q0DAJ3.2, DAA05097.1, BAF75367.1
LlWRKY14	OsWRKY55, 22, 19, 74, 71, 28, 32, 53	ABR25636.1, DAA05087.1, DAA05084.1, AAT84161.1, Q6QHD1.1, Q0DAJ3.2, DAA05097.1, BAF75367.1
LlWRKY15	OsWRKY10, 17, 26, 67	DAA05075.1, DAA05082.1, DAA05091.1, DAA05132.1
LlWRKY16	OsWRKY10, 17, 26, 67	DAA05075.1, DAA05082.1, DAA05091.1, DAA05132.1
LlWRKY17	OsWRKY55, 22, 19, 74, 71, 28, 32, 53	ABR25636.1, DAA05087.1, DAA05084.1, AAT84161.1, Q6QHD1.1, Q0DAJ3.2, DAA05097.1, BAF75367.1
LlWRKY18	OsWRKY68, 13, 39, 14	DAA05133.1 ,ABO34049.1,DAA05104.1,DAA05079.1
LlWRKY19	OsWRKY55, 22, 19, 74, 71, 28, 32, 53	ABR25636.1, DAA05087.1, DAA05084.1, AAT84161.1, Q6QHD1.1, Q0DAJ3.2, DAA05097.1, BAF75367.1
LlWRKY20	OsWRKY55, 22, 19, 74, 71, 28, 32, 53	ABR25636.1, DAA05087.1, DAA05084.1, AAT84161.1, Q6QHD1.1, Q0DAJ3.2, DAA05097.1, BAF75367.1
LlWRKY21	OsWRKY55, 22, 19, 74, 71, 28, 32, 53	ABR25636.1, DAA05087.1, DAA05084.1, AAT84161.1, Q6QHD1.1, Q0DAJ3.2, DAA05097.1, BAF75367.1
LlWRKY22	OsWRKY55, 22, 19, 74, 71, 28, 32, 53	ABR25636.1, DAA05087.1, DAA05084.1, AAT84161.1, Q6QHD1.1, Q0DAJ3.2, DAA05097.1, BAF75367.1

LlWRKY23	OsWRKY68,13,39,14	DAA05133.1 ,ABO34049.1,DAA05104.1,DAA05079.1
LlWRKY24	OsWRKY68,13,39,14	DAA05133.1 ,ABO34049.1,DAA05104.1,DAA05079.1
LlWRKY25	OsWRKY36	DAA05101.1
LlWRKY26	OsWRKY68,13,39,14	DAA05133.1, ABO34049.1, DAA05104.1, DAA05079.1
LlWRKY27	OsWRKY68,13,39,14	DAA05133.1 ,ABO34049.1,DAA05104.1,DAA05079.1
LlWRKY28	OsWRKY55, 22, 19, 74, 71, 28, 32, 53	ABR25636.1, DAA05087.1, DAA05084.1, AAT84161.1, Q6QHD1.1, Q0DAJ3.2, DAA05097.1, BAF75367.1
LlWRKY29	OsWRKY55, 22, 19, 74, 71, 28, 32, 53	ABR25636.1, DAA05087.1, DAA05084.1, AAT84161.1, Q6QHD1.1, Q0DAJ3.2, DAA05097.1, BAF75367.1
LlWRKY30	OsWRKY55, 22, 19, 74, 71, 28, 32, 53	ABR25636.1, DAA05087.1, DAA05084.1, AAT84161.1, Q6QHD1.1, Q0DAJ3.2, DAA05097.1, BAF75367.1
LlWRKY31	OsWRKY55, 22, 19, 74, 71, 28, 32, 53	ABR25636.1, DAA05087.1, DAA05084.1, AAT84161.1, Q6QHD1.1, Q0DAJ3.2, DAA05097.1, BAF75367.1 1
LlWRKY32	OsWRKY55, 22, 19, 74, 71, 28, 32, 53	ABR25636.1, DAA05087.1, DAA05084.1, AAT84161.1, Q6QHD1.1, Q0DAJ3.2, DAA05097.1, BAF75367.1
LlWRKY33	OsWRKY55, 22, 19, 74, 71, 28, 32, 53	ABR25636.1, DAA05087.1, DAA05084.1, AAT84161.1, Q6QHD1.1, Q0DAJ3.2, DAA05097.1, BAF75367.1
LlWRKY34	OsWRKY68, 13, 39, 14	DAA05133.1, ABO34049.1, DAA05104.1, DAA05079.1
LlWRKY35	OsWRKY68, 13, 39, 14	DAA05133.1, ABO34049.1, DAA05104.1, DAA05079.1
LlWRKY36	OsWRKY68, 13, 39, 14	DAA05133.1, ABO34049.1, DAA05104.1, DAA05079.1
LlWRKY37	OsWRKY55, 22, 19, 74, 71, 28, 32, 53	ABR25636.1, DAA05087.1, DAA05084.1, AAT84161.1, Q6QHD1.1, Q0DAJ3.2, DAA05097.1, BAF75367.1
LlWRKY38	OsWRKY55, 22, 19, 74, 71, 28, 32, 53	ABR25636.1, DAA05087.1, DAA05084.1, AAT84161.1, Q6QHD1.1, Q0DAJ3.2, DAA05097.1, BAF75367.1

Table S4: Primers of 16 *LlWRKY* genes for qRT-PCR

LlWRKY Genes	Forward primer (5'-3')	Reverse primer (5'-3')	Product Size (bp)
LlWRKY1	TGAGCTCACCGAAAACACTG	ATGGAGTGGAGTTTGGCTTG	176
LlWRKY2	AGCCAAAAAGCAAGTGGAGA	AGTGGGCTCTGCAGTTCAGT	177
LlWRKY3	GTTCGTGAACCAAGGGTTGT	GCGAGGCTCTTTCTATGTGC	175
LlWRKY4	GACCAAGCAGATGGAGAAGC	AGGTGATCTCTGGGACATGG	211
LlWRKY5	AGCAACCACCACTCCAATTC	TCGAACTCATTCCTCCCATC	220
LlWRKY6	ACAACCGAATGTCGTTAGCC	CTTGTTGTGCCTGCTGATGT	153
LlWRKY7	TTCAGTGAAGAAGCGTGTGG	TTGTTGGCCATTGTTGCTTA	202
LlWRKY8	GATCCTCAGCAGCCAAACTC	CCACTCCACCTTCCTCTTCA	171
LlWRKY9	GATACTGAATTGGGGGCAGA	TGCGCCATCTATAACCATCA	172
LlWRKY10	TCATACAGAGGGCAGCACAG	TGTTCCATCTTTGTCGTGGA	158
LlWRKY11	ATGGGATTCGAGTCTGGTTG	TGTGCTCTCCCTCGTAGGTT	191
LlWRKY12	TCAGATGCTCCTTTGCTCCT	ATAGGAGCGGACCCTTTGTT	196
LlWRKY13	CCTCTCTCCAGTCCGACAAG	ATCAGAGAATGGCCCATCAG	214
LlWRKY18	CAGCTTGGGATCCGAGAATA	CTTCCTCCACGACCACGTAT	190
LlWRKY20	GTGACGGTGGAGGCACTAAT	ATCTTCCTCTTGCTGCTGGA	242
LlWRKY22	ATGGATGCCAGTGGAGAAAG	GTGTGCCTTCGTATGTGGTG	162
Lactin	TGGTGTGATGGTTGGTATGG	TTTGCCTTAGGGTTGAGTGG	222

Table S5: Domain characteristics of the 38 full or partial LlWRKY proteins identified.

Sequence name	Conserve WRKY domain	Conserve zinc finger domain
LlWRKY1	WRKYGQK	CX4-CX23-HXH
LlWRKY2	WRKYGQK	CX5-CX23-HXH
LlWRKY3	WRKYGQK	CX4-CX22-HXH
LlWRKY4	WRKYGQK	CX4-CX22-HXH
LlWRKY5	WRKYGQK	CX4-CX23-HXH
LlWRKY6	WRKYGQK	CX4-CX23-HXH
LlWRKY7	WRKYGQK	CX4-CX23-HXH
LlWRKY8	WRKYGQK	CX4-CX23-HXH
LlWRKY9	WRKYGQK	CX4-CX23-HXH
LlWRKY10	WRKYGQK	CX4-CX23-HXH
LlWRKY11	WRKYGQK	CX5-CX23-HXH
LlWRKY12	WRKYGQK	CX5-CX23-HXH
LlWRKY13	WRKYGQK	CX5-CX23-HXH
LlWRKY14	WRKYGQK	**CX7-CX23-HXC**
LlWRKY15	**WRKYGKK**	CX4-CX23-HXH
LlWRKY16	**WRKYGKK**	CX4-CX23-HXH
LlWRKY17	WRKYGQK	**CX7-CX23-HXC**
LlWRKY18	WRKYGQK	CX5-CX23-HXH
LlWRKY19	WRKYGQK	**CX7-CX23-XXX**
LlWRKY20	WRKYGQK	**CX7-CX23-XXX**
LlWRKY21	WRKYGQK	**CX7-CX23-HXC**
LlWRKY22	WRKYGQK	CX5-CX23-HXH
LlWRKY23	WRKYGQK	CX5-CX23-HXH
LlWRKY24	WRKYGQK	CX5-CX23-HXH
LlWRKY25	WRKYGQK	CX5-CX23-HXH
LlWRKY26	- - - - - - QK	CX5-CX23-HXH
LlWRKY27	WRKYGQK	CX4-CX217/- - -
LlWRKY28	WRKYGQK	CX4-CX23-HXH
LlWRKY29	- - - - - - - - -	CX4-CX23-HXH
LlWRKY30	- - - - - - - - -	CX4-CX23-HXH
LlWRKY31	WRKYGQK	**CX7-CX23-HXC**
LlWRKY32	WRKYGQK	CX7-CX23-XXX
LlWRKY33	WRKYGQK	**CX7-CX23-HXC**
LlWRKY34	- - - - - - QK	CX5-CX23-HXH
LlWRKY35	- - - - YGQK	CX5-CX23-HXH
LlWRKY36	WRKYGQK	CX5-CX23-HXH
LlWRKY37	WRKYGQK	CX5-CX21/- - - -
LlWRKY38	WRKYGQK	**CX7-CX24-HXC**

Note: the dashed lines (- - -) denote missing sequences.

Table S6: The sequences of conservative lily WRKY motifs identified using the program MEME.

Sequence name	Conserve WRKY motif	Conserve zinc finger motif
LlWRKY1	DEHAWRKYGQKPIKGSPYPRGYYKCSSVK	RGYYKCSSVKGCPARKHVERALDDPTML VVTYEGEHY HAGQRELLGP
LlWRKY2	DSWSWRKYGQKPIKGSPYPRGYYKCSTSK	RGYYKCSTSKGCSAKKQVEKCRTDASVLI ITYTSSHN HPGPEFIHCP
LlWRKY3	DGYRWRKYGQKVVKGNPNPRSYYKCTSPG	RSYYKCTSPGCSVRKHIERASQDLQSVITT Y EGRHNHEVPP
LlWRKY4	DGYNWRKYGQKQVKGSEFPRSYYKCTHHN	RSYYKCTHHNCQVKKKVECNHEGHVPEI TYK GGHSHPKTPL
LlWRKY5	DGYNWRKYGQKQVKGSEFPRSYYKCTHPN	PRSYYKCTNPGCMVRKHVERASHDLKSV ITTYEGKHN HDVPAARNSN
LlWRKY6	DGYNWRKYGQKHVKGSEFPRSYYKCTHPN	PRSYYKCTNAGCPVRKHVERASNDPKAVI TTYEGKHN HDVPVARNIN
LlWRKY7	DGYRWRKYGQKAVKNSPHPRSYYRCTTQK CSVKKRVERS	RSYYRCTTQKCSVKKRVERSFEDPTIVITT Y EGQHNHHSPS
LlWRKY8	DGYEWRKYGQKYSRRISMNRSYFKCRNND	RSYFKCRNNDCSVKRKVEWDPSDPSNLRI VY DGTHNHPSPS
LlWRKY9	DGYNWRKYGQKQVKGSEYPRSYYKCTHPN	PRSYYKCTSVGCNVRKHVERASNDPKAVI TTYEGKHN HDVPAARNNS
LlWRKY10	DGYRWRKYGQKTVKGNPNPRSYYRCTYNG	RSYYRCTYNGCPVRKHVERASDDEKSILI SY EGKHNHDLPG
LlWRKY11	DRYEWRKYGQKVTRDNPSPRAYFKCSCAP	RAYFKCSCAPSCPVKKKVQRAAYDRSILI ATYEGEHN HGQPFSSTIG
LlWRKY12	DGYQWRKYGQKVTRGNPYPRAYFRCSFAP	RAYFRCSFAPSCAVKKKVQRSLQDRSILV ATYEGEHN HAHPYEKVAV
LlWRKY13	DGHQWRKYGQKVTKDNPCPRAYYRCSYAP	RAYYRCSYAPICPVKKKVQRSAEDRSILV ATYEGEHN HGHSHGAGVN
LlWRKY14	DGYSWRKYGQKEILGAKHPRGYYRCTHRN TRGCLATKQV	YYRCTHRNTRGCLATKQVQRSDQNPSILD ITYRGDHT CHQKPQLISP
LlWRKY15	DGFKWRKYGKKSVKNSPNPRNYYRCSSQD	RNYYRCSSQDCGVKKRVERDRDDARYV VTTY EGMHNHSSPD
LlWRKY16	DGFKWRKYGKKSVKNSPNPRNYYRCSSQD	RNYYRCSSQDCGVKKRVERDRDDARYV VTTY EGMHNHSSPD
LlWRKY17	DGHNWRKYGQKNIQYAKYPRCYYRCSYSE	YYRCSYSETRNCPARKQVQQSDENLSAFE ITYIQTHT CYPSEAHNCP
LlWRKY18	DTWSWRKYGQKPIKGSPYPRGYYRCSSAK	YYRCTHRNTRGCLATKQVQRSDQNPSILD ITYRGDHT CHQKPQLISP
LlWRKY19	DAYSWRKYGQKRIYNATFPRCYYRCTHKP	YRCTHKPDRGCQATRQVQQSEEDPAMFV STR ADPTRTKLLL
LlWRKY20	DAYSWRKYGQKRIYNATFPRCYYRCTHKP	YRCTHKPDRGCQATRQVQQSEEDPAMFV STR ADPTRTKLLL
LlWRKY21	DAYSWRKYGQKRIYKATFPRCYYRCTHKP	YYRCTHKPDRGCQATKQVQQSEEEPTMF VITYMGEHT CRSNPNKAST
LlWRKY22	DGCQWRKYGQKIAKGNPCPRAYYRCTVAQ	RAYYRCTVAQDCPVRKQVQRCAEDMSIL ITTYEGTHN HPLTISATAM
LlWRKY23	DLWAWRKYGQKPIKGSPYPRGYYRCSSLK	RGYYRCSSLKGCPARKQVERNPADPGLL MITYTAEHN HAVPTHRNSL
LlWRKY24	DLWAWRKYGQKPIKGSPYPRGYYRCSSLK	RGYYRCSSLKGCPARKQVERNPADPGLL MITYTAEHN HAVPTHRNSL
LlWRKY25	DRYEWRKYGQKVVKNTQHPRSYYRCVRDN	RSYYRCVRDNCRVKKRVERLAEDPRMVI TTY EGRHAHSPPR
LlWRKY26	- -	RAYYKCSTVRGCPARKFVELALDEPSVLI LNYEGEHH HANGAKNLRM
LlWRKY27	DGYEWRKYGQKYISSIGKNRSYFKCRDRN	- -

LlWRKY28	DGYRWRKYGQKVVKGNPNPRSYYKCTSVG	RSYYKCTSVGCPVRKHVERASNDLRAVITTY EGKHNHDVPP
LlWRKY29	- -	PRNYYRCSTNGCTVKKRVERDIDDPSYVINTYEGIHN HTSPSVVYYT
LlWRKY30	- -	TRNYFRCSTDGCSVKKRVERDTDDSSYVITTYEGTHN HTSPSVVYYT
LlWRKY31	DPHSWRKYGQKNIQYAKYPRCYYRCTYSE	YYRCTYSETRNCPAKKQVQQSDDNSSALKVTYIETHT CYSREVRNCH
LlWRKY32	DAYSWRKYGQKRIYNPTFPRCYYRCTHKP	YRCTHKPDRGCQATRQVQQSEEDPAMFVSTR ADPTRTKLLL
LlWRKY33	- -	YYRCTHKPDRGCQATRQVQQSEEDPAMFVITYMGEHT CRSGTTEPMT
LlWRKY34	- -	RGYYKCSTVRGCPARKFVEFALDEPSVLILTYEGVHH HAHGAKSLRT
LlWRKY35	- -	RGYYRCSSSKGCPARKQVERSRADPGVLVITYTAEHN HPVPTHRSSL
LlWRKY36	DLWAWRKYGQKPIKGSPYPRGYYRCSSLK	RGYYRCSSLKCPARKQVERNPADPGLLMITYTAEHN HAVPTHRNSL
LlWRKY37	DGCQWRKYGQKIAKGNPCPRAYYRCTVAP	AYYRCTVAPACPVRKQVQRCAEDMTILITTY E
LlWRKY38	DGHEWRKYGQKDIKGTVFPRCYYKCTYAD	- -

Note: the dashed lines (- - -) denote missing sequences.
