# Supplementary figures and images for: Genome-Wide Transcriptomic Identification and Functional Insight of Lily WRKY Genes Responding to Botrytis Fungal Disease
Source: Plants (Basel). 2021 Apr 15;10(4):776. doi: 10.3390/plants10040776 (PMC8071302; doi:10.3390/plants10040776)

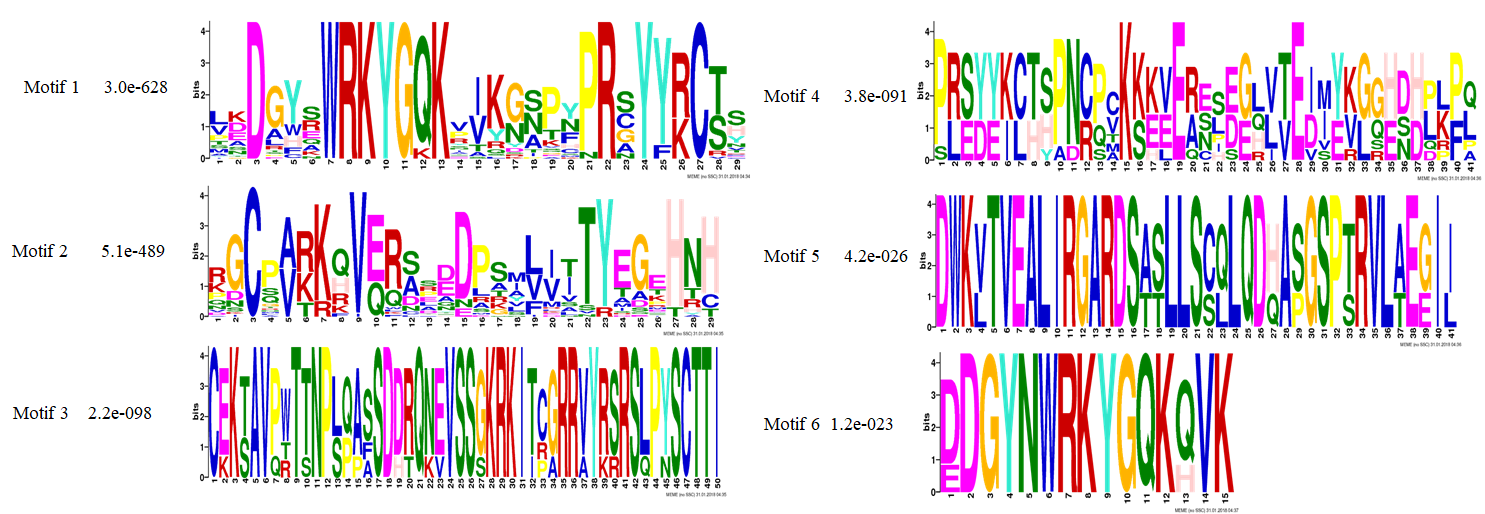

Supplement: Supplementary file 1 [file plants-10-00776-s001.zip › plants-1104701-supplementary/Figure S1-300dpi.tif]

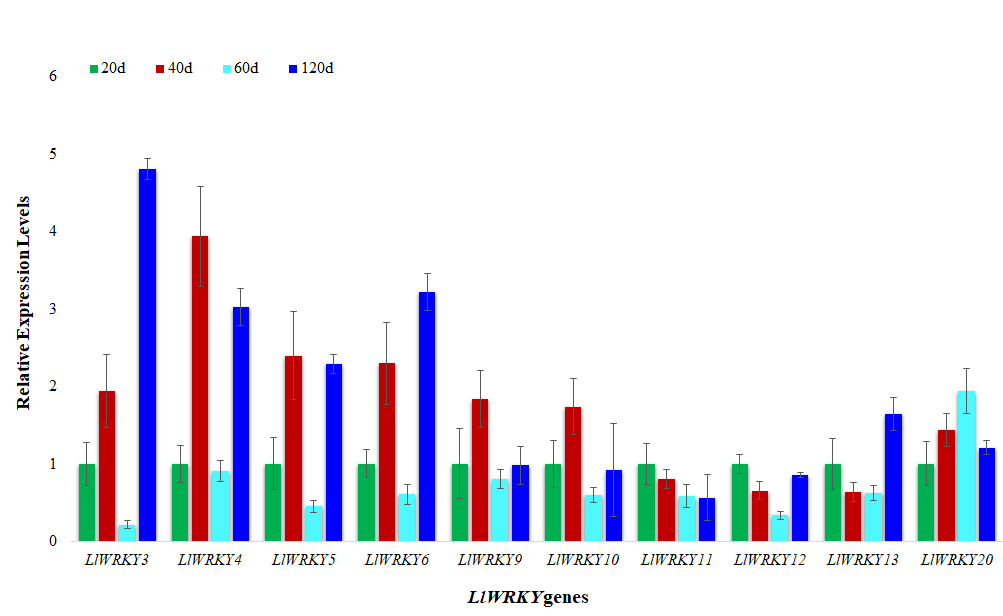

Supplement: Supplementary file 1 [file plants-10-00776-s001.zip › plants-1104701-supplementary/Figure S2-300dpi.tif]

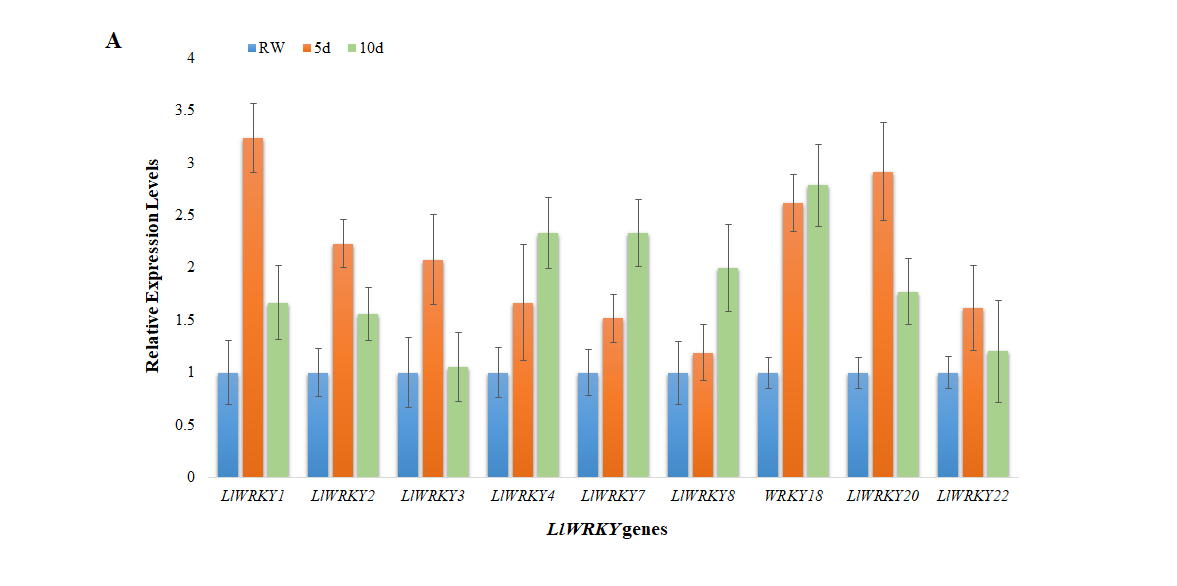

Supplement: Supplementary file 1 [file plants-10-00776-s001.zip › plants-1104701-supplementary/Figure S3A-300dpi.tif]

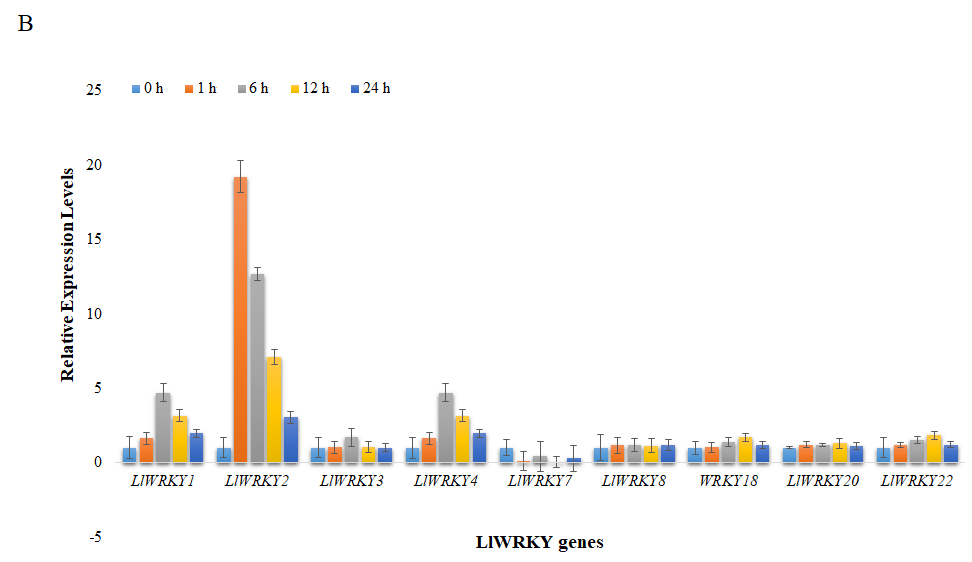

Supplement: Supplementary file 1 [file plants-10-00776-s001.zip › plants-1104701-supplementary/Figure S3B-300dpi.tif]

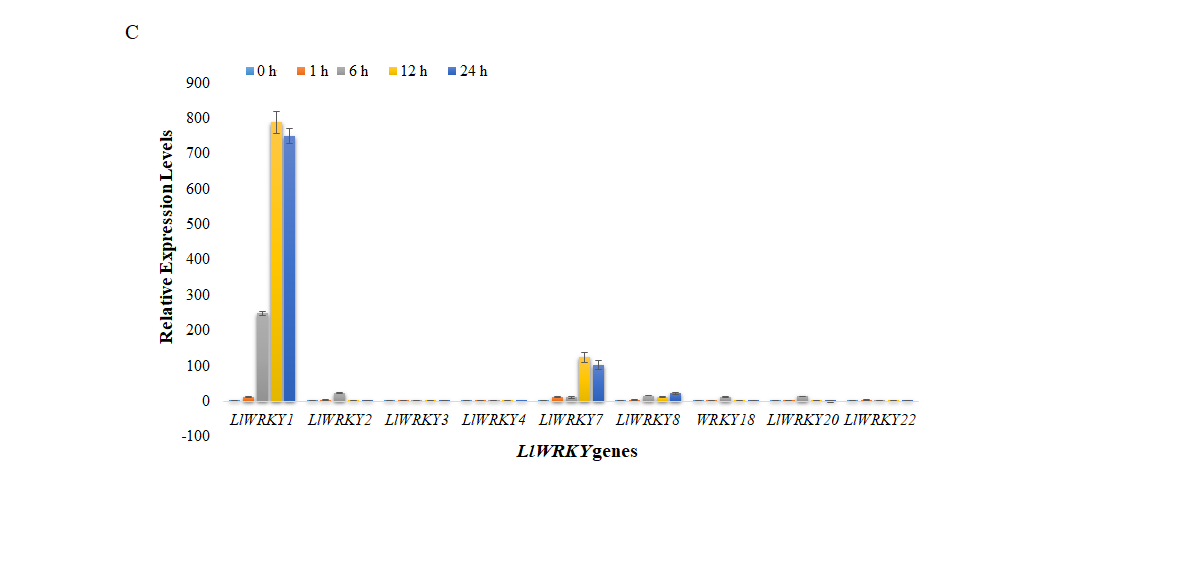

Supplement: Supplementary file 1 [file plants-10-00776-s001.zip › plants-1104701-supplementary/Figure S3C-300dpi.tif]

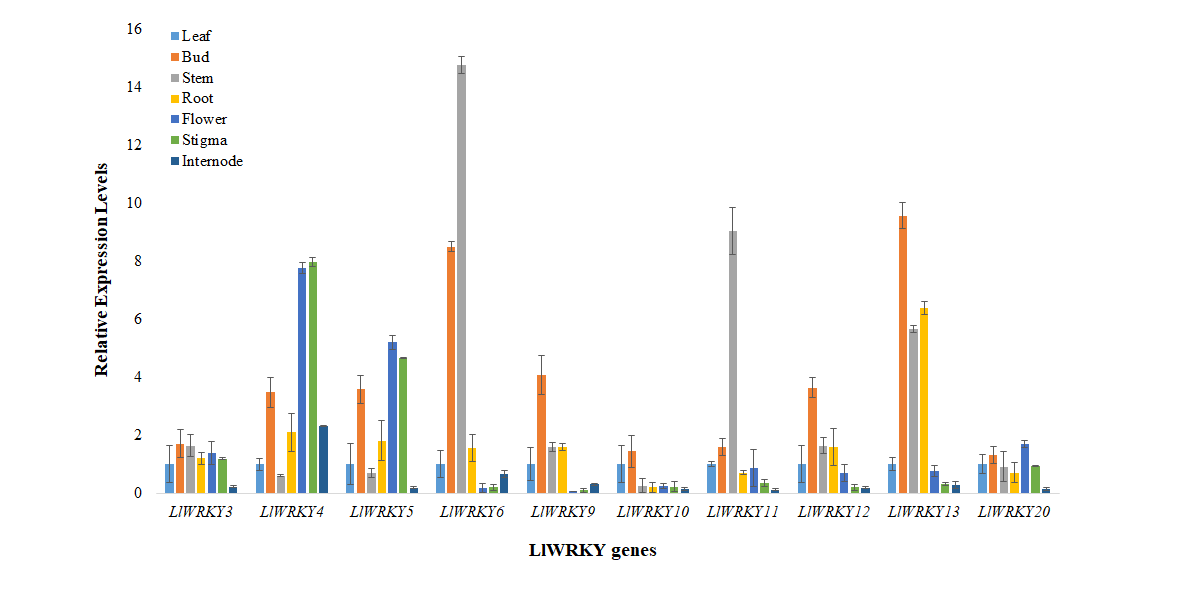

Supplement: Supplementary file 1 [file plants-10-00776-s001.zip › plants-1104701-supplementary/Figure S4-300dpi.tif]

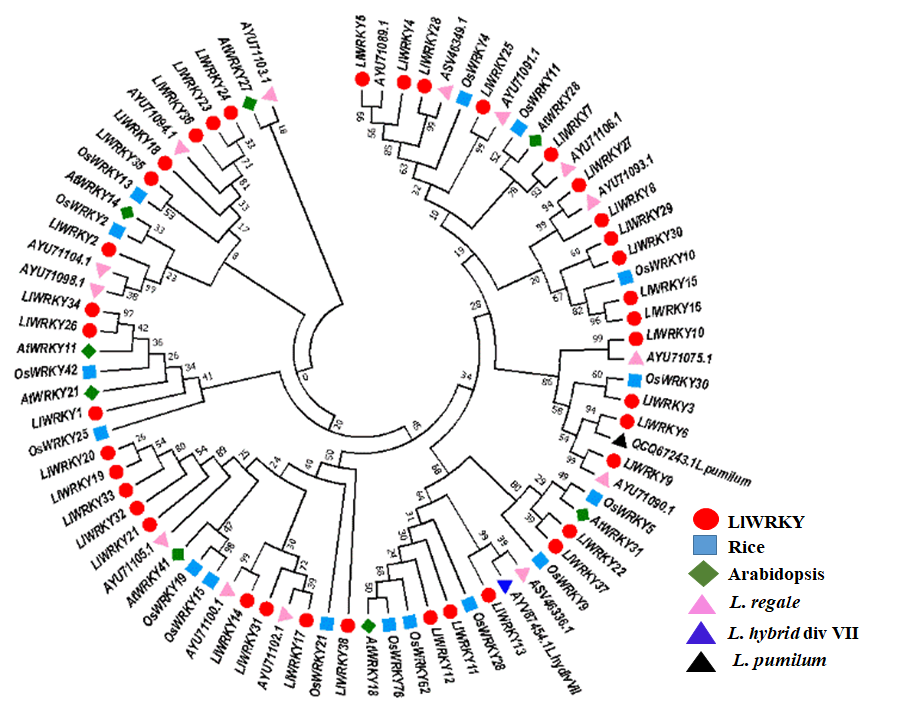

Supplement: Supplementary file 1 [file plants-10-00776-s001.zip › plants-1104701-supplementary/FigureS5-300dpi.tif]
